# Design and Fabrication of a Differential Electrostatic Accelerometer for Space-Station Testing of the Equivalence Principle

**DOI:** 10.3390/s16081262

**Published:** 2016-08-10

**Authors:** Fengtian Han, Tianyi Liu, Linlin Li, Qiuping Wu

**Affiliations:** Department of Precision Instrument, Tsinghua University, Beijing 100084, China; liuty14@mails.tsinghua.edu.cn (T.L.); lill09@163.com (L.L.); wuqiuping@mail.tsinghua.edu.cn (Q.W.)

**Keywords:** equivalence principle, differential accelerometer, electrostatic suspension, variable-capacitance motor, space station experiment, fundamental physics experiment

## Abstract

The differential electrostatic space accelerometer is an equivalence principle (EP) experiment instrument proposed to operate onboard China’s space station in the 2020s. It is designed to compare the spin-spin interaction between two rotating extended bodies and the Earth to a precision of 10^−12^, which is five orders of magnitude better than terrestrial experiment results to date. To achieve the targeted test accuracy, the sensitive space accelerometer will use the very soft space environment provided by a quasi-drag-free floating capsule and long-time observation of the free-fall mass motion for integration of the measurements over 20 orbits. In this work, we describe the design and capability of the differential accelerometer to test weak space acceleration. Modeling and simulation results of the electrostatic suspension and electrostatic motor are presented based on attainable space microgravity condition. Noise evaluation shows that the electrostatic actuation and residual non-gravitational acceleration are two major noise sources. The evaluated differential acceleration noise is 1.01 × 10^−9^ m/s^2^/Hz^1/2^ at the NEP signal frequency of 0.182 mHz, by neglecting small acceleration disturbances. The preliminary work on development of the first instrument prototype is introduced for on-ground technological assessments. This development has already confirmed several crucial fabrication processes and measurement techniques and it will open the way to the construction of the final differential space accelerometer.

## 1. Introduction

The equivalence principle (EP) is one of fundamental hypotheses of Einstein’s general relativity. The EP experiment is significant to verify the general relativity and search for new interactions or for new gravitational potential. The EP has been intensively tested by many experiments in a stated relative precision of several parts per 10^13^ [1,2], limited mainly by terrestrial environmental noises. Currently, several satellite- or balloon-based space experiment missions, such as the MicroSCOPE [3], STEP [4], GG [5], GreAT [6] and TEPO [7], are underway or proposed to test the equivalence to a precision better than 10^−15^–10^−18^. However, all of the above EP experiments tested the equivalence between non-spinning test masses. On the other hand, the gauge theory of gravitation with torsion predicts that a spin particle or a rotating extended body would deviate geodesic motion. In 2001, Zhang et al. developed a phenomenological model for the spin-spin interaction between rotating rigid spheres and suggested the idea for a space test of the new equivalence principle (NEP) [8]. A design case in which uses two concentric spherical shells with different rotations predicts a possible NEP violation on the order of 10^−14^. Based on the model in [8], a dimensionless parameter $\eta_{s}$, which would imply existence of spin-spin force between rotating masses and the Earth can be defined as:

(1) $$\eta_{s}=\frac{\Delta g}{g}=\kappa \left(\frac{{\vec{S}}_{A}\cdot {\vec{S}}_{e}}{Gm_{A}M_{e}R_{A}}-\frac{{\vec{S}}_{B}\cdot {\vec{S}}_{e}}{Gm_{B}M_{e}R_{B}}\right)$$

where *G* is the Newtonian gravitational constant, *m*_A_, *m*_B_, and *M*_e_ are the masses of two rotating bodies and the Earth, and ${\vec{S}}_{A}$, ${\vec{S}}_{B}$ and ${\vec{S}}_{e}$ are their spin angular momenta, *R*_A_ and *R*_B_ are the distances between the centers of the two masses and the Earth, respectively. The parameter κ represents the universal coupling factor for the spin-spin interaction with an upper limit of 3.4 × 10^−34^ kg^−1^ [8].

A series of double free-fall based terrestrial experiments using a rotating gyroscope and a non-rotating one showed that the spin-gravity interaction between the extended bodies was not observed at a relative accuracy of 1.6 × 10^−7^ [9,10]. The ground experiment is mainly limited by the friction of the mechanical gyroscopes, short duration of free fall and large seismic noises [11]. The experiment limits on the Earth have motivated a move to space. An indirect test for the NEP in space was from the data of GP-B space experiments, which gives indirectly the parameter $\eta_{s}\leq 10^{-11}$ [12]. A concept design of a double free-fall space NEP experiment onboard a drag-free satellite, in which two TMs made of the same material but rotated with much different angular velocity drop freely, was proposed to achieve an accuracy better than 10^−15^ [13]. However, this requires many challenging techniques to develop the NEP instrument, drag-free satellite, and extensive preflight testing to verify that the instrument performance can be reached once in space.

Recently, China has revealed that it will start building its first permanent space station which will be a T-shaped station consisting of a core capsule and two experimental capsules [14]. China’s space station will be fully operational around 2022 and then run on the orbit for more than 10 years. The NEP experiment is one of candidate space-station experiment missions in the fields of fundamental physics. For the first such experiment, it is designed to reach a measurement precision of 10^−12^ with an observation period of 20 orbits, which is five orders of magnitude better than the results obtained on the Earth. In this work, we present the design and preliminary development of a NEP experiment instrument, i.e., differential electrostatic accelerometer. The design of the sensor unit, modeling and simulation of electrostatic suspension in five degrees of freedom (DoFs) and spin control along the sensitive axis are described to evaluate the instrument performance. Considering the attainable micro-gravity condition provided by a quasi-drag-free capsule inside the space station, acceleration noise analysis of the NEP instrument is discussed to verify the NEP test goal. The development status of the first NEP instrument is presented for preliminary ground testing and proof of concept design.

This paper is organized as follows: in Section 2 we briefly introduce the concept design of the NEP experiment in the space station. The detailed design and noise evaluation of the NEP instrument are presented in Section 3. The preliminary work on developing an instrument prototype for ground testing is described in Section 4. Finally, Section 5 concludes this paper.

## 2. Concept of the Space Station-Based NEP Experiment

**Figure 1:**
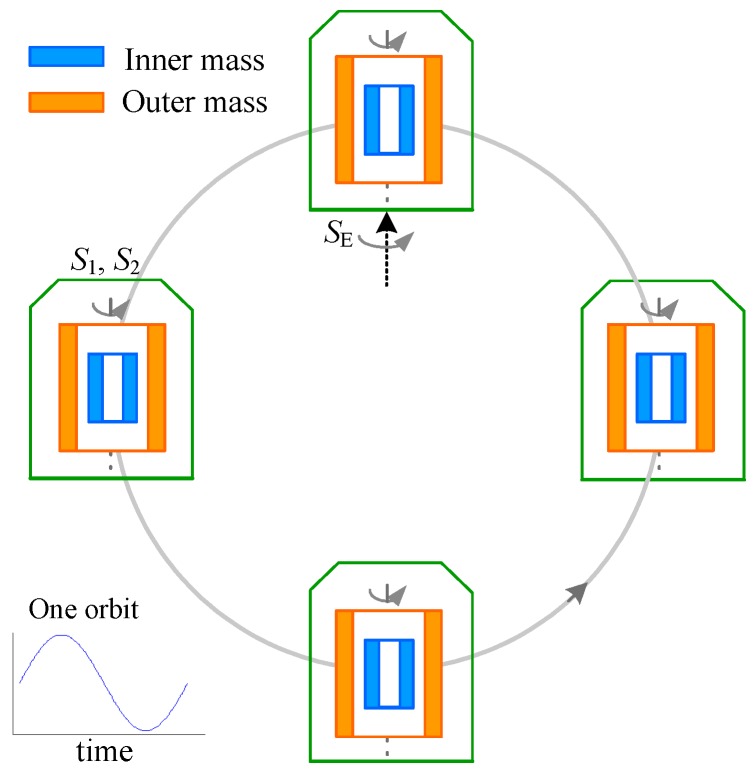
Concept of the space-station test of gravitational effects of two concentric rotating masses made of the same material. The cylindrical inner/outer test masses are suspended electrostatically in a differential accelerometer and spinning with much different angular momenta.

The concept of the NEP experiment is basically a free fall test which will use the low-noise space environment, long measurement duration, and a very precise experimental instrument to achieve the target precision of 10^−12^. The space station will provide a perfect comparison of the free falling of two rotating masses in the same significant gravity field over a long time span. Then, an observed nonzero value of $\eta_{s}$ would imply violation of the NEP or existence of spin-spin force between the rotating extended mass and the Earth. In the modified double free-fall space test, the two test masses (TMs) are maintained on the same trajectory and rotating with much different spin angular momenta, as shown schematically in Figure 1. A difference in the forces necessary to maintain the common trajectory will indicate the existence of the spin-spin force between the rotating mass and the Earth. The measurements will be made by placing the two masses inside a differential electrostatic accelerometers (used as the NEP instrument), where surrounding electrodes will apply weak forces to maintain the mass position fixed relative to the instrument frame. Two TMs have a common center of mass to be exposed to the same gravitational field, and have a carefully defined cylindrical form to provide the desired measurement accuracy. The sensitive axis of the NEP instrument which lies in the orbital plane is stabilized in a quasi-inertial pointing mode. The Earth gravity attraction is projected in a sine wave leading to the NEP violation signal detection at a known orbital frequency *f*_NEP_ and phase.

The attitude, thermal and atmospheric drag effects of the space station will be isolated by a low-pressure (~100 Pa) micro-gravity science experiment capsule inside which the NEP instrument will be accommodated. Similar to a drag-free spacecraft [15], the dedicated experiment capsule will be drag-compensated by the actuation of thrusters and virtually free floating inside the spacious space of the space station during NEP tests. It is expected to provide a microgravity level on the order of 10^−6^ m/s^2^ and a test duration up to 48 h. In this way, the experiment capsule will follow the two TMs in their almost purely gravitational motion and the in-orbit motions of the two TMs, maintained as free as possible, could be finely compared to estimate any NEP violation signal. The experiment capsule will carry one differential accelerometer compatible with a room temperature experiment. The entire space experiment mission will last over six months including initial verification of the instrument, in-orbit calibration and final NEP tests.

The ultra-sensitive differential accelerometer is crucial to the NEP test precision. The electrostatic suspension provides the capability to generate very weak but accurate force, allowing the accelerometers to achieve extremely high resolution by largely decreasing their measurement ranges [16]. During the instrument operation, both masses are controlled with respect to the same instrument frame: the sum of these forces is maintained virtually null benefiting from good microgravity level provided by the floating experiment capsule. The NEP instrument provides a precise, low noise measurement of the difference in acceleration of the two masses to search for the NEP violating signal. For example, by considering the Earth gravity acceleration at an orbit altitude of 400 km, i.e., 8.69 m/s^2^, the NEP test can be performed at an accuracy better than 10^−12^ by attaining a differential acceleration resolution of 8 × 10^−12^ m/s^2^. Considering a measurement integrated over 20 orbits, corresponding to 1.1 × 10^5^ s, the required measurement noise along the sensitive axis is 2.65 × 10^−9^ m/s^2^/Hz^1/2^ in the vicinity of the frequency *f*_NEP_ = 0.182 mHz. In our design goal, the allowed total noise for the NEP instrument operated in space station environment is limited to a spectral density less than 2.0 × 10^−9^ m/s^2^/Hz^1/2^ at *f*_NEP_.

## 3. Design and Simulation of the NEP Instrument

The NEP instrument configuration is similar to the space accelerometer for gravitation experimentation (SAGE) developed for the on-orbit MicroSCOPE mission [17]. In this weak equivalent principle (WEP) test, free fall motion of two non-spinning test masses which are composed of different materials is observed when both masses are subjected exactly to the same gravitational field. In comparison to the MicroSCOPE mission [3], the proposed NEP experiment aims to verify the spin-spin interaction between rotating extended bodies and the Earth. Hence, two TMs of the differential NEP accelerometer are made of the same material but rotated with much different angular momenta. The most challenging aspect in design of the NEP instrument is that the two TMs are spinning during on-orbit tests, such as 10^4^ rpm for the inner TM and 100 rpm for the outer TM. During operation there is no any physical contact on the TMs to enable continuous rotation of the inner and outer masses. In this case, the thin gold wire scheme commonly used in traditional space accelerometers, which enables the position detection and permits the control of the electric charge of the mass [18], is not applicable to the NEP accelerometer. Therefore, the NEP instrument design is significantly different from previous EP instruments, such as SAGE. A comparison among the NEP instrument and two similar inertial sensors are listed in Table 1. The continuous rotation of the two TMs makes the NEP instrument design more complicated, especially in the sensor unit and rotation control electronics.

**Figure 2:**
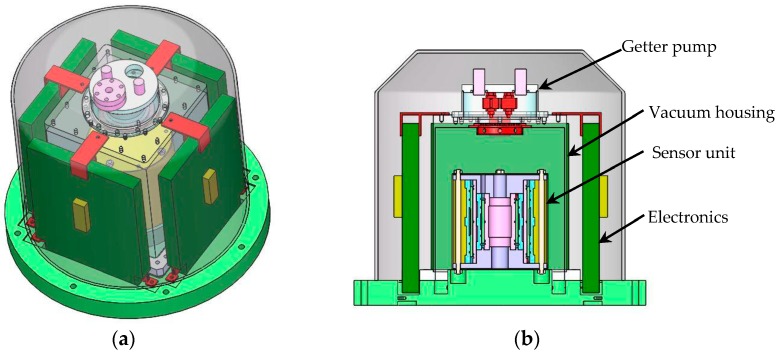
NEP instrument: (**a**) Mechanical sensor core mounted in a tight housing with above the vacuum system and surrounding the suspension and rotation control units; (**b**) Cross-sectional view of the differential accelerometer including two concentric test masses.

Unlike those WEP space experiments using two or more onboard differential accelerometers for comparison [3,4], the NEP experiment will need only one differential accelerometers for the space station-based EP mission. The design parameters should be compatible with the microgravity environment provided by the floating experiment capsule. A conceptual design of the NEP instrument shown in Figure 2 has a profile dimension Ф280 mm × 240 mm. The sensor unit is composed of two concentric electrostatic accelerometers in which each TM is suspended and centered by five-axis electrostatic suspension. The initial spinning-up of the mass around the sensitive axis is based on the principle of a variable capacitance motor [21]. The sensor core consisting of two TMs and four electrode cylinders will be fixed in a tight vacuum housing to reduce the radiometer effect and gas fluctuation dissipation.

### 3.1. Sensor Unit

**Figure 3:**
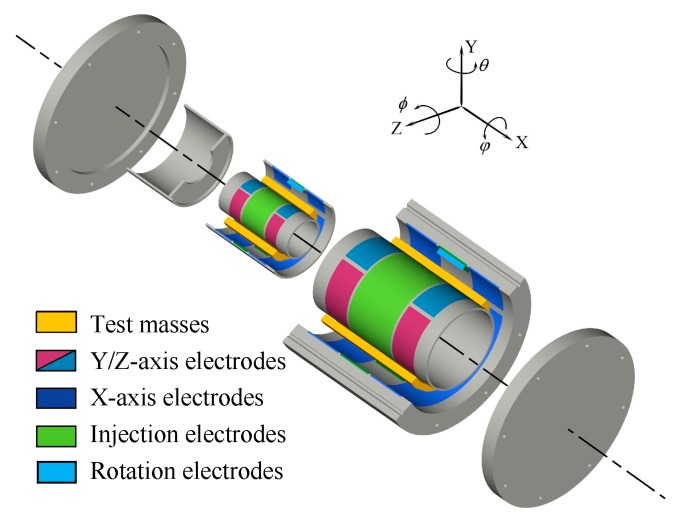
Exploded view of the differential accelerometer core. The inner and outer test masses are aligned concentrically and coaxially. The sensitive axis of the NEP test is along the *x* direction.

This differential accelerometer is specifically optimized for the space test of the NEP. The mechanical core of the NEP instrument contains two concentric inertial sensors, each composed of one hollow quasi-cylindrical TM and two concentric electrode cylinders which are positioned within and without the mass, as shown in Figure 3. The material of the two masses has been selected to titanium alloy by taking into account of the purity, specific gravity, homogeneity, machining ability, thermal stability, electrical conductivity and magnetic susceptibility. Given that the two TMs are not idealized point masses, a common center of mass is not sufficient to guarantee both bodies will be subjected to the same force. Therefore, the cylinder dimensions should be carefully designed to approximate the monopole property and thus reduce the effects of local gravity gradient fluctuations [17,22]. This effect can be minimized by design of the TM dimensions with equal moments of inertia on each axis, which has been carefully considered and adopted by several WEP missions [3,4]. Moreover, to further minimize the gravity gradient effects on the NEP signal, the off-centering between the two TMs is expected to be less than 20 μm after the instrument fabrication and integration [3]. The two TMs are initially designed by modeling and optimization of overall suspension and rotation performances, as listed in Table 2.

**Figure 4:**
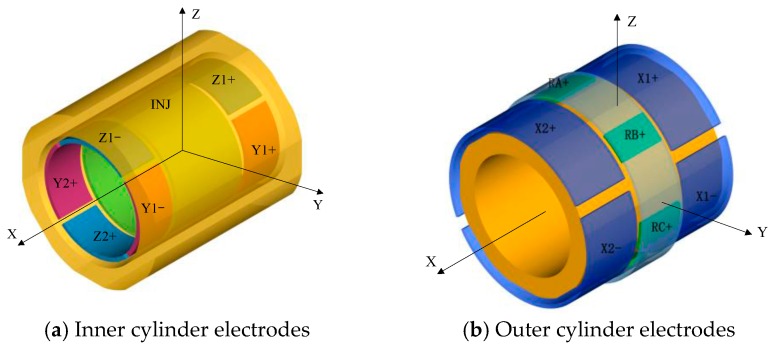
Electrode configuration of the NEP instrument with a cylindrical test mass (in orange): (**a**) On the inner cylinder are four quadrant electrodes on the vertical axis for *z* and *θ*, four electrodes on the horizontal axis for *y* and *ϕ*, and one injection electrode for capacitive position sensing; (**b**) On the outer cylinder are the *x* electrodes around the entire circumference, as well as a ring of six stator electrodes for rotation control around the *x* axis.

Each test mass is surrounded by two gold coated cylinders made of ultra-low expansion (ULE) glass ceramic, exhibiting a 1.6 × 10^−7^/°C coefficient of thermal expansion at 25 °C. Associated with high thermal stability of the instrument interfaces, it ensures a very steady set of electrical conductors around the mass [3]. The necessary electrodes on two cylinders are defined by gold coating to function as capacitive position sensing, electrostatic suspension and rotation control of the mass, as depicted in Figure 4. As the electrostatic force is always attractive, the actuation electrodes act in pairs to provide both a positive and a negative force for any degree of freedom. On the inner cylinder, there are four quadrant electrodes (Z_1+_, Z_1−_, Z_2+_, Z_2−_) for the radial *z*- and ϕ-axes suspension and other four quadrant electrodes (Y_1+_, Y_1−_, Y_2+_, Y_2−_) for the radial *y*- and *θ*-axes, respectively. Moreover, to enable the capacitive position detection, a sinusoidal excitation voltage is applied to the TM via one common injection electrode (INJ). On the outer cylinder, there are four axial electrodes (X_1+_, X_1−_, X_2+_, X_2−_) around the entire circumference, as well as a ring of six stator electrodes for rotation control around the *x* direction. The rotation operation around the *x*-axis is based on the principle of variable-capacitance electrostatic motors [21]. Note that the *x*-axis accelerometer provides the most sensitive measurements, which are utilized to test gravitational effects of the spinning mass. Therefore, along the axial sensitive direction, the configuration is such that the gradients of capacitances between the electrodes and the mass are quite independent of the position of the TM, which doesn’t induce the first-order electrostatic negative stiffness [22]. Likewise, the corresponding geometry parameters of the four electrode cylinders are listed in Table 3. The four cylinders of the two inertial sensors will be integrated on a unique ULE centering part, serving as a reference frame for the NEP instrument. 

### 3.2. Electrostatic Suspension

**Figure 5:**
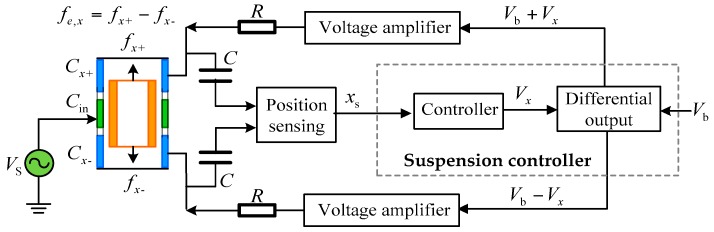
Schematic sketch of the *x*-axis electrostatic suspension loop. Five loops similar to this one are required to suspend each test mass electrostatically.

This electrostatic accelerometer is based on the force-balance technology which measures the electrostatic force necessary to maintain the TM motionless with respect to the sensor cage [22]. The motion of each TM with respect to highly stable instrument frame is actively servo-controlled by electrostatic suspension in five DoFs: translations along the *x*, *y* and *z* axes, and rotations around two in-plane axe, *θ* and *ϕ*, respectively. Figure 5 shows a schematic diagram of the electrostatic suspension loop for the *x* axis. A sinusoidal carrier signal $V_{S}$ (~100 kHz) is applied to the injection electrode which is utilized to capacitively couple the excitation signal to the TM for capacitive position sensing [23]. In the presence of external force, the TM displaces away from its nominal position, resulting in a change in the differential capacitance, $C_{x1}-C_{x2}$, between the TM and a pair of top and bottom suspension electrodes. The position sensor senses this capacitance change and then feeds the position signal $x_{s}$ into a feedback controller to stabilize the suspension servo loop. In order to linearize the electrostatic feedback force, the suspension system is normally operated in a differential fashion so that the voltage on the positive electrode is the sum of a bias voltage $V_{b}$ and a control voltage $V_{x}$ while the voltage on the negative electrode is produced by subtracting the bias voltage from the control voltage. Finally, the feedback voltages from two voltage amplifiers are applied on the suspension electrodes by which the the electrostatic force is generated to balance the external force, pulling the TM back to its nominal position. The resultant feedback forces are derived from the accurate measurement of the applied voltages on the pairs of electrodes. Note that these suspension electrodes function in pairs to simultaneously measure and control the TM position, with the complete set providing contactless electrostatic suspension in five DoFs. The RC networks shown in Figure 5 are used to separate relatively low frequency forcing voltages from high frequency position sensing signals.

Adequate suspension stiffness and precise centering control of the TM are important to ensure desirable performance of the force-balanced electrostatic accelerometer. To stabilize the movement of each TM, we need to control its motion in five DoFs. If all the cross-coupling effects among the different axes are ignored by assuming small position deviation, the dynamics of the TM can be modeled by five uncoupled 1-DoF systems:

(2a) $$m{\ddot{e}}_{i}=f_{e,i}+f_{\mathrm{in},i}$$

(2b) $$J_{j}{\ddot{\alpha }}_{j}=M_{e,i}+M_{\mathrm{in},i}$$

where m and J are the mass and moment of inertia of the TM, e and α the linear and angular displacements of the TM away from its equilibrium position, $f_{e}$ and $M_{e}$ the electrostatic feedback force and torque, $f_{\mathrm{in}}$ and $M_{\mathrm{in}}$ the externally applied input force and torque, the subscript i=x,y,z denotes the axis along which the force is produced and j=θ,ϕ denotes the axis around which the torque is produced, respectively. The residual air-film damping effect is neglected in Equation (2) by considering that the TM is suspended in high vacuum.

For instance, the electric force produced by the charged set of the feedback electrodes on the mass along the considered *x*-axis can be linearized as [24]:

(3) $$f_{e,x}=k_{v}V_{x}+k_{x}x$$

where $k_{v}$ is the actuator gain and $k_{x}$ the position stiffness or negative spring constant, respectively. The maximum electric force applied on the centered TM is $k_{v}V_{b}$ by considering the condition $\left|V_{x}\right|\leq V_{b}$ holds. Note that the *x*-axis suspension design is optimized to generate weak electrostatic force by using an area variation scheme [18]. In principle, it is free of the negative position stiffness, i.e., $k_{x}=0$. On the other hand, the position stiffness has a significant effect on other four suspension axes in which a gap variation scheme is utilized to generate relatively large electrostatic force in order to balance possible disturbance resulted from the TM spinning.

This linear model is valid by assuming that the motion of the PM is very small, e.g., in a range less than 10% of the nominal gap. This assumption can be ensured by adequate suspension stiffness provided through design of the suspension control loop. Substituting Equation (3) into Equation (2a) and then taking the Laplace transform yield the dynamics of the TM:

(4) $$\frac{X(s)}{F_{\mathrm{in},x}(s)+k_{v}V_{x}(s)}=\frac{1}{ms^{2}-k_{x}}$$

where $F_{\mathrm{in},x}(s)+k_{v}V_{x}(s)$ represents the total forces applied on the TM along the *x*-direction. The dynamics of the TM in four other DoFs can be found by substituting appropriate variables in Equation (4). 

**Figure 6:**
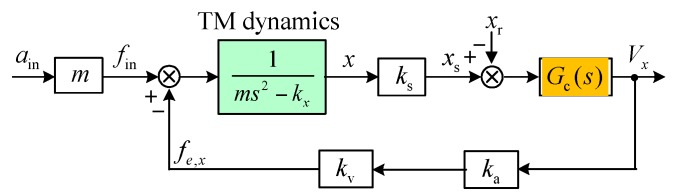
Block diagram of the closed-loop suspension control system used as a force-balanced electrostatic accelerometer: *x*_r_ and *x*_s_ are the position reference input and position sensor output used to evaluate the loop frequency response as defined in Equation (6).

Open loop instability due to the negative position stiffness can be found by inspecting the characteristic equation of the transfer function in Equation (4) given the presence of a pole on the right-hand side of the s-plane. Consequently, active feedback control must be used to stabilize the suspension servo loop. A simplified block diagram of the closed-loop suspension control is shown in Figure 6, where $k_{s}$, $k_{a}$ and $G_{c}(s)$ are the sensitivity of the position sensor, gain of the voltage amplifier and transfer function of the feedback controller to be designed, respectively. It is assumed that no significant time delay (phase lag) occurs in the position sensor, digital controller and drive amplifier in the frequency range of the instrument operation.

For the NEP instrument operating in high vacuum, a typical lag-lead compensator is utilized to illustrate the design procedure:

(5) $$G_{c}(s)=k_{c}\frac{\left(1+T_{1}s\right)}{\left(1+aT_{1}s\right)}\frac{\left(1+T_{2}s\right)}{\left(1+T_{2}s/b\right)},\;a>1,\;b>1,\;T_{1}>>T_{2}$$

where $k_{c}$ is the compensator gain selected according to the stiffness requirements, $T_{1}$ and $T_{2}$ denote the time constants of the lag and lead compensators, respectively.

The closed-loop transfer function from the position reference input $x_{r}$ to the position sensor output $x_{s}$ is used as a measure of the loop dynamic performance and is given by:

(6) $$G_{\mathrm{cl}}(s)=\frac{X_{s}(s)}{X_{r}(s)}=\frac{k_{s}k_{a}k_{v}G_{c}(s)}{ms^{2}-k_{x}+k_{s}k_{a}k_{v}G_{c}(s)}$$

Equation (6) describes the frequency response of the suspension servo to the position input. On other hand, the feedback control voltage is a measure of the acceleration input *a*_in_ applied to the instrument case. The transfer function that describes the accelerometer input-output response is defined as:

(7) $$G_{a}(s)=\frac{V_{x}(s)}{A_{\mathrm{in}}(s)}=\frac{m}{k_{v}}G_{\mathrm{cl}}(s)$$

Equation (7) shows that the static and dynamic performance is closely related to design of the suspension control loop. At frequencies much lower than the suspension loop bandwidth, the TM is kept motionless in the electrode cavity due to high loop gain. In this case, Equation (7) can be simplified to a scale factor $G_{a}(s)\approx K_{a}=m/k_{v}$ by considering that $G_{\mathrm{cl}}(s)\approx 1$.

The electrostatic suspension loop is basically a regulator that keeps the TM centered at its equilibrium position. One of the most important characteristics of the accelerometer is the electrostatic stiffness provided by feedback control. The stiffness transfer function of the closed-loop system that relates the equivalent acceleration input force $ma_{\mathrm{in}}$ to the position change x is derived from Figure 6 and given by:

(8) $$K(s)=\frac{mA_{\mathrm{in}}(s)}{X(s)}=ms^{2}-k_{x}+k_{s}k_{a}k_{v}G_{c}(s)$$

A fundamental controller design criteria for the suspension loop is to provide adequate stiffness and damping for stable suspension of the TM. The suspension stiffness is largely determined by the feedback controller within the loop bandwidth [24]. Closed-loop suspension control must provide adequate positive stiffness, which is greater than the inherent negative position stiffness $k_{x}$ to ensure loop stability. Hence, the suspension design must satisfy the following condition:

(9) $$k_{c}>n\cdot ak_{x}/k_{a}k_{v}k_{s},\;n>1$$

where *n* is the ratio of the lowest overall stiffness to the negative stiffness of the TM and is usually *n* ≥ 5 by design.

Given that the residual air-film effect in high vacuum can be neglected in design of the suspension loop, the lead compensation is utilized to provide adequate damping force and ensure desirable stability. The compensated open-loop transfer function in Figure 6 can be given by:

(10) $$G_{\mathrm{ol}}(s)=\frac{k_{s}k_{a}k_{v}G_{c}(s)}{ms^{2}-k_{x}}$$

It will be helpful to relate the lead compensator parameters in Equation (5) to the cross-over frequency of the open-loop system, e.g., let $\omega_{c}=\sqrt{b}/T_{2}$. Substituting *s* = j*ω* into Equation (10), the cross-over frequency can be obtained by definition, i.e., ${\left|G_{\mathrm{ol}}(s)\right|}_{s=j\omega_{c}}=1$:

(11) $$\omega_{c}=\sqrt{\frac{(n\sqrt{b}-1)k_{x}}{m}}$$

where *n* can be set by stiffness requirement and *b* for desirable phase margin. The time constant of the lead compensator can be derived as $T_{2}=\sqrt{b}/\omega_{c}$ while the lag compensator usually has a time constant setting that satisfies $T_{1}\geq 3T_{2}$ and a≥10.

**Figure 7:**
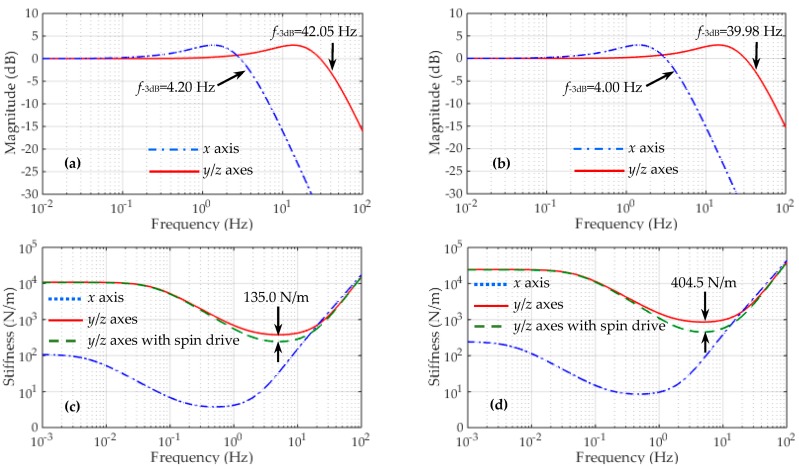
Simulation results of the active suspension control loops for the inner TM (left side) and the outer TM (right side): (**a**,**b**) Closed-loop frequency responses; (**c**,**d**) Suspension stiffness of the closed-loop control system.

The design parameters of the *x*, *y*, *z*-axis suspension are listed in Table 4, where the *y*/*z* position stiffness also contains the contributions from the *x*, *y*, *z-*axis suspension electrodes and the injection electrode. The closed-loop frequency responses of the differential accelerometer operated in vacuum are simulated based on Equation (6). The simulation results of totally six closed-loop suspension loops, three for the inner TM and three for the outer TM, are shown in Figure 7. It is clear that much different closed-loop bandwidths between the *x*-axis and the *y*/*z-*axis suspension loops are shown in Figure 7a,b. This result is attributed to the measuring range in the *y* and *z* axes of the inner/outer accelerometers being over 50 times larger than that in the sensitive *x* axis.

Frequency responses of the suspension stiffness based on Equation (8) are shown in Figure 7c,d. The simulated curves clearly indicate that these suspension loops have similar stiffness characteristics, except that the two radial (*y*/*z*) suspension loops have much higher stiffness than the axial (*x*) loop in the low-frequency and medium-frequency ranges. On the other hand, these curves coincide closely in the high-frequency range (>200 Hz). This result can be explained by considering Equation (8) that each accelerometer has an identical inertial stiffness, *mω*^2^. A design consideration in these suspension loops is that the stiffness characteristics must match the full-scale range of the accelerometer input. As an example, since the allowable input range in the radial direction is over 50 times larger than that in the sensitive *x* direction, it can be observed that the radial suspension has extremely high stiffness compared with that of the axial suspension. At a frequency of 10^−3^ Hz, Figure 7 indicates that the *y*/*z*-axis stiffness is 100 times higher than that of the *x*-axis suspension in our design. These results confirm the optimization of the instrument configuration for the *x* axis as the measurement axis for the NEP test. The small full-scale range and low stiffness make it the most sensitive axis for the NEP measurements.

It seems that the closed-loop system exhibits relatively high suspension stiffness by setting a larger *n* (*n* = 15). For instance, the allowable displacement of the inner TM is less than 1.72 μm, only 2.87% of the nominal gap. However, the spin drive voltage during the electrostatic motor operation will also generate large unstable position stiffness [25], e.g., 135.0 N/m by the inner motor and 404.5 N/m by the outer motor at a drive voltage of 100 V. In this case, the allowable motion range of the inner TM is increased to 2.67 μm but also well below the design goal of 10 μm. Considering that the unstable motor stiffness is over five times larger than the negative position stiffness *k_x_*, its effect on the loop stability should be taken into account in design of the controller gain, as in Equation (9). In our design by setting *n* = 15, the lowest closed-loop stiffness is over two times higher than the total negative spring constant, which ensures the loop stability even during the initial spin-up process.

### 3.3. Electrostatic Motor

**Figure 8:**
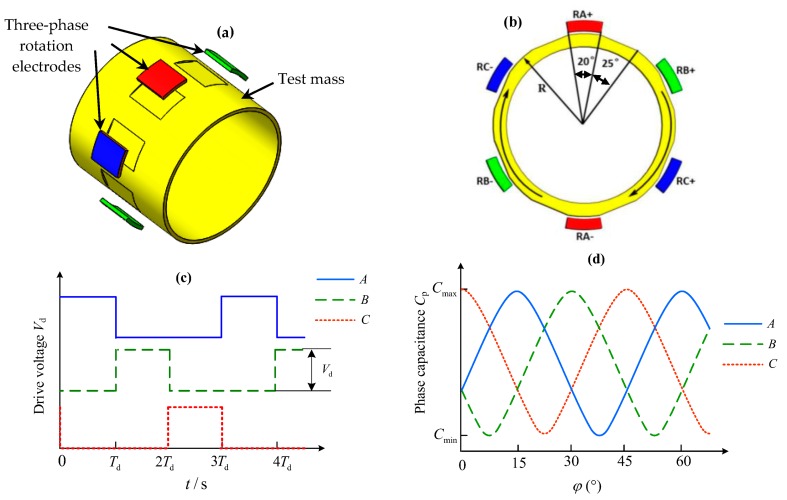
Three-phase variable-capacitance motor: (**a**) Stator electrodes and test mass (rotor); (**b**) Layout of the 6-electrode stator and 8-pole rotor; (**c**) Three-phase excitation voltages and (**d**) Three-phase stator-rotor capacitances as a function of the mechanical rotation angle.

One important feature of the NEP instrument is that the two test masses are spinning with much different rotations. For this space accelerometer, each TM also features eight flat areas on its external surface as depicted in Figure 8a, allowing the measurement and control of the axial mass spin. The rotation of the mass around the sensitive axis is measured though the differential capacitance change between the dedicated flat areas on the mass and associated rotation electrodes on the outer cylinder [21]. The spinning-up of the mass is based on principle of the variable capacitance motor (VCM) consisting of a 6-pole stator (six rotation electrodes) and an 8-pole rotor (test mass). This VCM operates in a three-phase drive mode and produces a step angle of 15°, as shown in Figure 8.

In order to simulate the steady-state speed and start-up time responses of the electrostatic motor, an analytical model governing the rotation behavior is necessary. For this three-phase VCM operated in high vacuum, the dynamic equation describing the TM rotation around the *x*-axis can be expressed as:

(12) $$J_{x}\dot{\omega }+b_{x}\omega =T_{e}$$

where $J_{x}$ and $b_{x}$ are the axial moment of inertia of the mass and the damping coefficient generated by the residual gas in vacuum cavity, and $T_{e}$ the drive torque produced by three-phase rotation electrodes.

**Figure 9:**
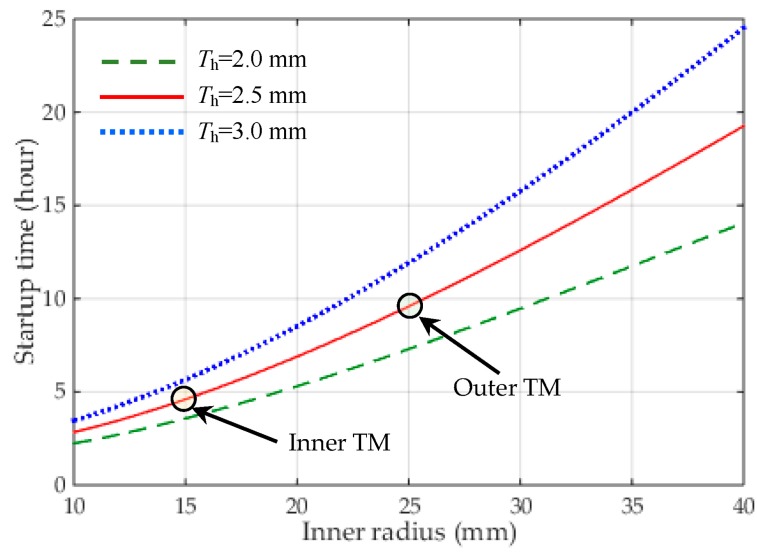
The start-up time of the motor required to spin up from 0 to its rate speed of 10^4^ rpm vs. the inner radius of the TM. The thickness of the cylindrical mass *T*_h_ is set at 2.0, 2.5 and 3.0 mm for comparison.

Although the NEP instrument measurements are quite insensitive to the effects of electrostatic drive by comparing with classical electromagnetic motors, the drive torque produced by the electrostatic motor is extremely weak. Therefore, the start-up duration for a non-spinning mass to its rated speeds, 10^4^ or 10^2^ rpm in this work, will be quite long. Assuming the drive voltage is set at 100 V and the ultra-high vacuum reaches 10^−5^ Pa, Figure 9 shows the start-up time responses of the motor with different geometry parameters of the spinning TM. For instance, the start-up process from 0 to 10^4^ rpm will last 1.657 × 10^4^ s (4.603 h) for the inner mass and 3.452 × 10^4^ s (9.588 h) for the outer mass, respectively. Fortunately, once the TM reaches its desired speed, the rotation drive will be switched off to avoid any possible disturbing forces produced by the rotation electrodes. Then the two TMs will be keeping free rotation virtually at their rated speeds. The small speed decay during each NEP experiment can be measured by a capacitive speed sensor and thus used to assist the NEP experiment data processing. Although longer time is needed to spin up the TM, the VCM drive is compatible with electrostatic suspension design and well suited for long-term space test of the NEP.

The NEP instrument can be set at several basic operation modes for calibration or NEP test, by considering that the two TMs can spin at various speed settings. When the two TMs are spinning at an equal speed, the instrument will be used as a reference to estimate various experimental limitations or systematic errors. On the other hand, in the NEP tests the two masses will be spinning at much different speeds, e.g., 10^4^ rpm for the inner mass and 100 rpm for the outer mass and vice versa. Hence, unlike the STEP or MicroSCOPE mission enclosing two or more differential instruments made of different materials, the NEP tests can be performed using only one differential accelerometer with multiple rotation modes.

### 3.4. Noise Analysis

As stated in Section 2, the desired acceleration noise along the sensitive axis is less than 2.0 × 10^−9^ m/s^2^/Hz^1/2^ in order to test the NEP at a level of 10^−12^. The resolution of the NEP instrument is limited by various noise sources. The noise performance of the NEP instrument comprising of both the inner and outer accelerometers will be evaluated considering the following four major noise sources.

**Figure 10:**
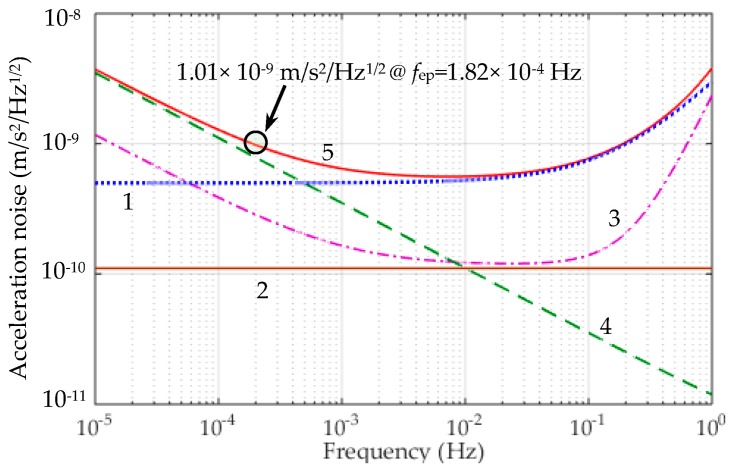
Acceleration noise budget for the sensitive axis of the NEP instrument. The lines 1, 2, 3, 4 and 5 represent the differential acceleration coupling from the residual non-gravitational acceleration, temperature effect, position sensing induced noise, electrostatic actuation noise and total NEP instrument noise, respectively.

First, the differential acceleration between the TMs coupling from the residual non-gravitational acceleration *a*_ec_ acting on the NEP experiment capsule can be written as:

(13) $$a_{d}=a_{\mathrm{ec}}/R_{\mathrm{cmr}}$$

where *R*_cmr_ is the common mode rejection ratio of the differential accelerometer along the sensitive axis. In the NEP space tests, a drag-free control system with micro-Newtonian thrusters will be used to compensate for the non-gravitational forces acting on the experiment capsule, such as atmospheric drag force. It is estimated that the residual non-gravitational acceleration could be reduced down to 5 × 10^−6^ × (1 + *f*/0.1) m/s^2^/Hz^1/2^. According to the requirement of the MicroSCOPE mission, the *R*_cmr_ could be realized to be 10^4^. Thus the acceleration disturbance *a*_d_ is estimated to be lower than 5 × 10^−10^ m/s^2^/Hz^1/2^, as shown by line 1 in Figure 10.

Next, the thermal instability *δT*_d_ of the instrument core induces radiation pressure and radiometer effects due to the residual gas pressure *P* inside the tight housing [7]. The temperature effect including the thermal radiation pressure and radiometer acceleration can be expressed as:

(14) $$a_{\mathrm{tem}}=a_{\mathrm{Radiometer}}+a_{\mathrm{Rad}-\mathrm{Pres}}\approx \frac{PA_{m}}{2Tm}\delta T_{d}+\frac{16\sigma A_{m}T^{3}}{3mc}\delta T_{d}$$

where *σ*, *c* and *A*_m_ are the Stefan-Boltzmann constant, light speed and section area of the TM, respectively. Given the residual gas pressure *P* = 10^−5^ Pa, temperature *T* = 300 K, *δT*_d_ = 0.5 K/Hz^1/2^, the resulting acceleration disturbance for the inner and outer TMs are 1.27 × 10^−10^ m/s^2^/Hz^1/2^ and 8.22 × 10^−11^ m/s^2^/Hz^1/2^, respectively. The total temperature effect *a*_tem_ is 1.51 × 10^−10^ m/s^2^/Hz^1/2^, as shown by line 2 in Figure 10.

At frequency range higher than 0.076 Hz, the position sensing noise *x*_noise_ affects the acceleration resolution with a square frequency law, i.e.,

(15) $$a_{\mathrm{Position}}=(4\pi^{2}f^{2}+\omega_{p}^{2})x_{\mathrm{noise}}$$

where $\omega_{p}=\sqrt{k_{x}/m}$ is the frequency associated to the residual passive stiffness *k*_x_ between the TM and associated suspension electrodes, which is evaluated to be less than 0.1 N/m by considering electrostatic field boundary effects. If the capacitive position sensor noise can be expressed as *x*_noise_ = 4 × 10^−11^ × (1 + 10^−3^/*f*)^1/2^ m/Hz^1/2^ [26], the total acceleration noise *a*_Position_ is less than 2.95 × 10^−10^ m/s^2^/Hz^1/2^ at *f*_NEP_, as shown in line 3 in Figure 10.

At frequency range lower than 10^−3^ Hz, the electrostatic actuation acceleration noise is the major source of noise and given by:

(16) $$a_{\mathrm{ea}}=\frac{k_{v}}{m}v_{\mathrm{noise}}$$

which depends mainly on the noise of the voltage amplifier *v*_noise_. In [27], the voltage noise is estimated as *v*_noise_ = 1.2 × 10^−8^ × *V*_b_ × (1 + 7/*f*)^1/2^ V/Hz^1/2^. Considering the actuator gain *k*_v_ and bias voltage *V*_b_ in Table 4, the voltage amplifier will contribute a total acceleration noise of 8.18 × 10^−10^ m/s^2^/Hz^1/2^ at *f*_ep_, as shown in line 4 in Figure 10.

Other accelerometer noise sources [7], such as the thermal noise induced by the residual gas damping, coupling between the capsule’s attitude and the relative position of the two TMs, suspension induced electrical noise due to the spinning TM, the Earth’s gravity gradient effect and magnetic effect, have also been considered for the noise evaluation. These effects are in the range of over two orders of magnitude less than the NEP instrument noise goal, i.e., below 2 × 10^−11^ m/s^2^/Hz^1/2^ at *f*_NEP_. Thus these noise sources are negligible in the noise evaluation.

Taking the square root of the quadratic sum of four considered acceleration noises, the total differential acceleration noise is shown by line 5 in Figure 10. The evaluated noise is 1.01 × 10^−9^ m/s^2^/Hz^1/2^ at the NEP signal frequency, by neglecting other small acceleration disturbances. It is clear that the electrostatic actuation and residual non-gravitational acceleration are two major noise sources at the NEP signal frequency.

## 4. Development Status of a NEP Instrument Prototype

A single cylindrical accelerometer prototype is proposed to demonstrate its technological feasibility. This section presents the configuration and design performance of the first NEP instrument prototype, presently developed by the authors at Tsinghua University.

### 4.1. The Sensor Unit

**Figure 11:**
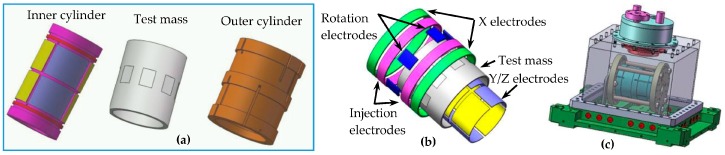
The sensor unit configuration: (**a**) Test mass and inner/outer cylinders; (**b**) Exploded view of various electrodes; (**c**) The assembled senor unit operated inside a vacuum chamber.

This prototype has similar dimensions to the inner cylindrical accelerometer of the differential NEP instrument. However, very high voltage needs to be generated in order to counteract the 1 g Earth’s gravity along the *y*/*z* axes [28]. To lower the required suspension voltage, the injection electrode is moved from the inner cylinder to the outer one so as to provide larger radial suspension electrode areas. The full-scale range of the *y*/*z* acceleration inputs is set at 3.0 g by applying a high suspension voltage of 786 V. Figure 11 shows the basic configuration of the sensor unit. The material chosen for the TM is an ultra-low thermal expansion glass ceramic, which is lighter than titanium alloy and thus easier to suspend under 1 g. The inner and outer cylinders are also made of the identical glass ceramic for better temperature stability. The necessary electrodes for electrostatic suspension and electrostatic motor are defined by gold coatings. To achieve low noise along the optimized *x* axis during ground tests, this sensitive axis is placed inside the horizontal plane and set at a much small input range of only 10^−4^ g.

**Figure 12:**
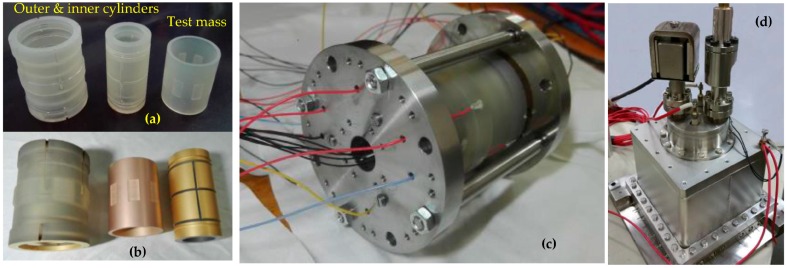
The fabricated sensor unit: core parts before (**a**) and after (**b**) gold-coating; (**c**) Assembled sensor unit core; (**d**) Sensor unit mounted inside a vacuum chamber.

From the present design of the sensor core, the production of the first laboratory model was completed and the instrument integration is now in progress. To achieve the challenging NEP test accuracy, the geometry of the core parts of the accelerometer, i.e., the electrode cylinders and the test mass, must be machined with an accuracy of a few micrometers in order to guarantee the fine electrostatic actuation of the instrument. Thus, the performance of the NEP mission relies dramatically on the machining and precise metrology of the parts of the sensor core and particularly on the test mass. Figure 12 shows three core parts and the fabricated sensor unit. These ULE parts are fabricated using a specific ultrasonic machining and precise grinding process. After fabrication, these core parts have undergone extensive measurements. The form and dimension measurements were performed using a coordinate measuring machine. From these measurements, all necessary features (diameters, length, concentricity, cylindricity, parallelism and perpendicularity) are determined. It is shown that the TM has a mass of 25.898 g, inner diameter 29.9986 mm, and outer diameter 35.0070 mm, where the dimensional tolerances are less than 7 μm while the geometrical tolerances are all less than 0.8 μm. Moreover, the capacitive gaps between the hollow TM cylinder and two inner/outer electrode cylinders are adjusted and calibrated by measuring the electrode capacitances using a precise LCR meter. The measured result shows that the mean gap is 45.7 μm and the gap variation throughout the entire sensitive axis is less than 0.6 μm. In addition, the vacuum chamber can be maintained at 2 × 10^−5^ Pa by a miniaturized sputtering ion pump.

### 4.2. The Sensing and Control Electronics

**Figure 13:**
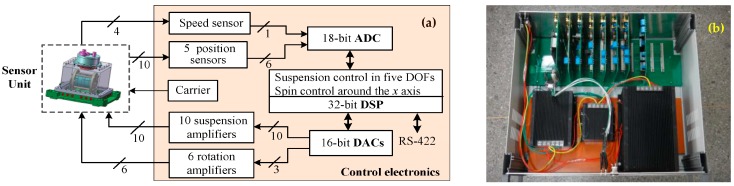
(**a**) Schematic of electrostatic suspension and rotation control electronics; (**b**) A photograph of the assembled control electronics unit.

Evaluating the performance of electrostatic accelerometers on the ground is inevitably limited by Earth’s 1 g gravity. To meet the needs of the electrostatic levitation, a high voltage amplifier-based suspension scheme is suitable to test the engineering and flight models of accelerometers for space missions, even though the resolution of the accelerometers could not be directly verified at the designed level due to the seismic noise and the high-voltage coupling limits [28]. Figure 13a illustrates the block diagram of the accelerometer electronics mainly consist of six capacitive position sensors, 16 high-voltage amplifiers and a 32-bit control-optimized digital signal processor (DSP), as well as the necessary ADCs and DACs. Five capacitive position sensors and ten high-voltage suspension amplifiers are used to maintain the TM at its equilibrium position by active electrostatic suspension in five DoFs. The spin angle of the TM is detected by a capacitive speed sensor and continuous rotation is then realized by driving six high-voltage rotation amplifiers synchronously. Figure 13b shows the suspension and rotation control electronics developed for subsequent ground testing of the NEP instrument. The measured results show that the capacitive sensor has a sensitivity of 0.45 V/pF and a bandwidth of 11.2 KHz. The gain of the high-voltage dc amplifier circuit is about 46 V/V and the output voltage range is from 0 to 920 V. The experimental frequency response shows a large signal bandwidth of 8.5 kHz. Considering the simulated closed-loop bandwidth in the radial (*y*/*z*) suspension loops is 1.11 kHz, the sampling frequency of the DSP-based digital controller (TMS320F28335) is set at 20 kHz. The simulation results shows the radial suspension loops exhibit much higher bandwidth and suspension stiffness than the sensitive axis owing to much different full-scale measuring ranges. Moreover, the simulated start-up time of the TM from 0 to 10^4^ rpm is 36.9 min by applying a high drive voltage of 300 V.

In this ground-test mode, the instrument functionality and performance will be evaluated as much as possible. According to current design and simulation results of the NEP instrument prototype during 1 g operation, the main design specifications are summarized in Table 5.

## 5. Conclusions

A space station based free-fall test of the equivalence principle with two rotating test masses is proposed to test the spin-gravity interaction at a precision of 10^−12^. We present the design of the differential electrostatic accelerometer specially optimized for the in-orbit test of the NEP and the noise evaluation to perform such a sensitive experiment on board a microgravity experiment capsule. This is more than five orders of magnitude better than the results performed on ground with free-fall rotating gyroscopes. This requires many challenging techniques to develop the NEP instrument, as well as in-orbit calibration and fine data processing. The configuration and fabrication of a single cylindrical accelerometer prototype are presented for ground testing and proof of concept. Extensive effort has been carried out to develop the means for accurate fabrication, assembly and measurements of the core components. The completed development of the sensitive NEP instrument is extremely challenging and time-consuming. Current work has already confirmed some technological assessments and it should continue to open the way to the final construction of a differential accelerometer. Future work will focus on electrostatic suspension, rotation control, calibration and ground testing of the accelerometer prototype. Most of these innovative technologies will be tested and verified in a series of ground-based pre-flight experiments. 

## Figures and Tables

**Table 1:** Comparison of three inertial sensors for space-based fundamental physics experiments (NA: not applicable).

Description	MicroSCOPE [3,19]	GP-B [20]	NEP
Inertial sensor	Differential accelerometer	Dual-axis gyroscope	Differential accelerometer
Measured signal	Differential acceleration	Spin axis drift	Differential acceleration
Acceleration noise	≤2.0 × 10^−12^ m/s^2^/Hz^1/2^	NA	≤2.0 × 10^−9^ m/s^2^/Hz^1/2^
EP signal frequency	0.17 mHz or 1 mHz	NA	0.182 mHz
Test mass	Two concentric cylinders	One solid sphere	Two concentric cylinders
Suspension	Each TM in six DoFs	In three DoFs	Each TM in five DoFs
Spinning up	NA	helium gas flow	Electrostatic motor
Spin rate (rpm)	0	3738–4962 (Measured)	10,000 (By design)
Sinusoidal excitation	Thin gold wire	Three phase excitation	By injection electrode
Charge control	Thin gold wire	Ultraviolet discharge	Ultraviolet discharge
Space platform	Drag-free satellite	Drag-free satellite	quasi-drag-free capsule floating inside space station

**Table 2:** Physical properties of the two test masses.

Description	Symbol	Inner TM	Outer TM
Material	--	Titanium alloy	
Mass (g)	*m*	43.92	110.10
Moment of inertia (kg∙m^2^)	*J*	1.105 × 10^−5^	6.890 × 10^−5^
Inner radius (mm)	*r*	15	25
Outer radius (mm)	*R*	17.5	27.5
Length (mm)	*l*	39.92	64.37
Radial nominal gap (μm)	*d*_0_	60	60
Rated spin speed (rpm)	*n*_0_	10,000	100

**Table 3:** Parameters of the electrode cylinders.

Description	Inner Accelerometer		Outer Accelerometer	
	Inner Electrodes	Outer Electrodes	Inner Electrodes	Outer Electrodes
Cylinder radius (mm)	14.94	17.56	24.94	27.56
Electrode separation (mm)	1.5	1.5	1.5	1.5
*y*/*z* electrode length (mm)	8	--	12	--
*x* electrode length (mm)	--	10	--	10
Injection electrode length (mm)	15.92	--	32.37	--
Rotation electrode length (mm)	--	26.92	--	51.4

**Table 4:** Parameters of the electrostatic suspension loops.

Parameter (unit)	Symbol	Inner TM		Outer TM	
		*y/z*	*x*	*y/z*	*x*
Bias voltage (V)	*V*_b_	20	20	20	20
Full-scale range (m/s^2^)	*A*_in,max_	1.48 × 10^−^^2^	2.96 × 10^−^^4^	1.48 × 10^−^^2^	1.85 × 10^−^^4^
Actuator gain (N/V)	*k*_v_	3.26 × 10^−^^5^	6.50 × 10^−^^7^	8.16 × 10^−^^5^	1.02 × 10^−^^6^
Position stiffness (N/m)	*k_x_*	24.14	0	54.71	0
Position sensor gain (V/m)	*k*_s_	10^6^	5×10^5^	10^6^	5 × 10^5^
Voltage driver gain (V/V)	*k*_a_	4	4	4	4
Ratio in stiffness design	*n*	15	--	15	--
Controller gain	*k*_c_	83.34	83.61	75.41	120.62
Cross-over frequency (Hz)	*ω*_c_	24.75	2.48	23.53	2.35

**Table 5:** Summary of main design specifications of the NEP instrument prototype.

Description (Unit)	*x*-Axis	*y/z*-Axis
Acceleration measuring range (g)	10^−4^	3.0
Scale factor (V/g)	10^5^	3.3
Acceleration noise at 0.1 Hz (m/s^2^/Hz^1/2^)	10^−7^	--
Suspension loop bandwidth (Hz)	10.7	1110.0
Suspension stiffness (N/m)	>19.5	>1.58 × 10^5^
Rated spin speed (rpm)	10^4^	--
Start-up time (min)	36.9	--
